# Essential Roles of E3 Ubiquitin Ligases in p53 Regulation

**DOI:** 10.3390/ijms18020442

**Published:** 2017-02-17

**Authors:** Sanam Sane, Khosrow Rezvani

**Affiliations:** Division of Basic Biomedical Sciences, Sanford School of Medicine, The University of South Dakota, Vermillion, SD 57069, USA; Sanam.sane@usd.edu

**Keywords:** p53 tumor suppressor protein, E3 ubiquitin ligases, tumor, cancer therapy, apoptosis

## Abstract

The ubiquitination pathway and proteasomal degradation machinery dominantly regulate p53 tumor suppressor protein stability, localization, and functions in both normal and cancerous cells. Selective E3 ubiquitin ligases dominantly regulate protein levels and activities of p53 in a large range of physiological conditions and in response to cellular changes induced by exogenous and endogenous stresses. The regulation of p53’s functions by E3 ubiquitin ligases is a complex process that can lead to positive or negative regulation of p53 protein in a context- and cell type-dependent manner. Accessory proteins bind and modulate E3 ubiquitin ligases, adding yet another layer of regulatory control for p53 and its downstream functions. This review provides a comprehensive understanding of p53 regulation by selective E3 ubiquitin ligases and their potential to be considered as a new class of biomarkers and therapeutic targets in diverse types of cancers.

## 1. Introduction

In mammalian cells, p53 protein is at the hub of a plethora of regulatory and signaling pathways that are crucial for cell-fate control. With 393 amino acids, p53 protein contains a complex set of domains. p53 has a transactivation domain (TAD) located at the N-terminus of protein that is further subdivided into two homologous subdomains, TAD1 (residues 1–40) and TAD2 (residues 41–61), followed by a proline-rich region (residues 63–94) [1]. The central domain is a DNA-binding domain (residues 94–292) which allows p53 to bind double-stranded target DNA. The variable binding affinity of p53 to response elements in promoters of p53 target genes allows p53 the specificity required for its functions [2]. The DNA-binding domain includes most of the cancer mutations found in human tumors [3]. The C-terminal region includes two domains: (1) The tetramerization domain (residues 325–356), which allows p53 to form a reversible tetramer structure. The presence of the tetramerization domain is essential for p53 binding to DNA and proteins as well as p53’s post-translational modifications and degradation [4]; (2) The C-terminal negative regulatory domain (CTD) of p53 is a natively unfolded region. CTD binds to single-stranded DNA (at the DNA end site) in contrast to the DNA-binding domain, which binds to the internal DNA site [5].

The transcriptional activity of p53 is important for several classical functions of p53 such as senescence, cell-cycle progression, apoptosis, and DNA repair. However, the tumor suppressor function of p53 goes beyond the above classical functions activated during DNA damage or hyperproliferative signals. Metabolic reprogramming, autophagy, stemness, invasion, and metastasis as well as communication within the tumor microenvironment are other cellular processes that are modulated by p53 transcription-dependent and -independent functions [6]. In addition to tumor suppression in cancer cells, recent studies underscore p53’s critical roles in normal homeostasis, metabolism, fertility, and differentiation in normal cells [7]. Subcellular localization of p53 is one of the critical factors that balances the tumor suppressor functions of p53 in non-cancerous cells versus cancerous cells [8]. Nuclear localization of p53 increases upon stress [9]. Phosphorylation of sites at the N-terminus of p53 and inhibition of murine double minute (Mdm)2 are two key factors in this nucleocytoplasmic translocalization [10].

An extreme set of regulations is necessary to control p53 under physiological and pathological conditions. Posttranslational modifications (PTMs) play a central role in regulating the tumor suppressor functions of p53 [11]. PTMs such as N-terminus phosphorylation of p53 positively affect p53’s tumor suppressor functions following stresses [11]. On the other hand, particular PTM-types are rewired in cancer cells to antagonize the tumor suppressor functions of wild-type (WT) p53 during tumor progression. In fact, suppression of p53’s functions through PTMs commonly happens in approximately 50% of cancer patients who retain WT-p53. The other half of cancers harbor p53 mutations that eliminate p53’s normal functions [12].

The development of strategies that can restore the tumor suppression and apoptotic functions of WT-p53 is a key therapeutic goal [13]. Increased levels of active WT-p53 tumor suppressor gene expression in cancer are particularly important for determining chemotherapeutic agent efficacy [14,15]. Despite numerous biological and chemical strategies focused on p53 recovery in tumor cells, the current anti-cancer drugs induces WT-p53 through DNA damage mechanisms. As a result, patients face several unwanted and often life-threatening side effects associated with these cytotoxic agents. Therefore, it is critical that we develop treatments for non-genotoxic restoration of WT-p53 function in human tumors in a more selective and tolerable manner. This review focuses on four E3 ubiquitin ligases that selectively and dominantly target p53 in tumors. We will systematically explain the molecular mechanisms by which p53 activity is controlled by these selected E3 ubiquitin ligases and their potential for developing into effective drug regimens.

## 2. Murine Double Minute (Mdm)2/Mdm4, a Dominant E3 Ligase of p53

E3 ubiquitin-protein ligase Mdm2 is the master negative regulator of the p53 tumor suppressor protein. In normal cells, the presence of Mdm2 is necessary to maintain the p53 protein at the basal level by regulating its ubiquitination and degradation by the 26S proteasome. Upon cellular stresses, the binding affinity of Mdm2 to p53 significantly decreases, resulting in p53 stabilization [16,17]. Mdm2 is also a transcriptional target of p53, creating a feedback loop that regulates both the levels and activity of p53 protein as well as expression of Mdm2 [18]. Besides ubiquitination and proteasomal degradation of p53 protein, Mdm2 inhibits p53’s transcriptional activity, an essential tool in the tumor suppressor functions of p53 [19,20]. Mdm2 has a p53 binding domain in the N-terminus and a RING domain in the C-terminus that functions as an E3 ubiquitin ligase. Binding the N-terminal of Mdm2 to p53 protein impedes the transcriptional activities of p53 [20]. Simultaneously, the C-terminus RING domain with intrinsic ubiquitin E3 ligase activity enables Mdm2 to recruit E2 ubiquitin-conjugating enzyme(s), which facilitate p53 ubiquitination and degradation by the proteasome complex [21]. Mdm2-dependent ubiquitination of p53 is a complex process, since low levels of Mdm2 are responsible for monoubiquitination and nuclear export of p53, while high levels of Mdm2 mediate polyubiquitination of p53 for nuclear degradation [20,22,23]. Overexpression of Mdm2 turns this E3 ubiquitin ligase into an oncoprotein in both p53-dependent and p53-independent manners [24,25]. Tumorigenic functions of overexpressed Mdm2 facilitate tumor metastasis and progressive forms of the disease in many human tumors including osteosarcomas, breast, prostate, and colon cancers [26,27,28,29,30,31,32]. Mdm2 and the therapeutic benefits of its inhibition have turned Mdm2 into a potential targeted therapy [33]. Several small-molecule Mdm2 inhibitors are under intense study by many labs or have entered clinical trial, such as nutilin-3 [34,35,36,37]. A better understanding of the regulation of Mdm2 is essential to developing novel and more effective cancer therapeutic strategies that can target Mdm2 in cancer cells while leaving normal cells intact [38,39].

As a main negative regulator of p53 [40], Mdm2 is fine-tuned by a group of protein partners that bind to Mdm2 in a context- and cellular-dependent manner. One of these protein partners is Mdm4 (Mouse double minute 4 or Mdmx) [41]. Mdm4 is an Mdm2 homolog with a RING domain located in the N-terminus of protein. Mdm4 oligomerizes to and increases Mdm2’s E3 ligase activity [42,43]. Oligomerization of Mdm2/Mdm4 enables Mdm2 to more efficiently regulate p53 activity [44]. Therefore, antagonists that are able to simultaneously inhibit both Mdm2/Mdm4 could have higher therapeutic success [45]. The regulatory roles of the Mdm2/Mdm4 complex during p53 turnover is further altered by two other E3 ligases, constitutive photomorphogenesis protein (COP1) and p53-induced protein with a RING-H2 domain (Pirh2). The interplay among Mdm2, Mdm4, Pirh2, and COP1 blocks ubiquitination of Mdm2 by Mdm4 and inhibits Mdm2 self-ubiquitination. In addition, these two E3 ubiquitin ligases synergistically function with the Mdm2/Mdm4 complex to inhibit p53’s activities, including p53-mediated transcriptional activity [46].

ARF (alternative reading frame) tumor suppressor protein (p14^ARF^) is an alternate reading frame protein product of the CDKN2A locus. The p14^ARF^ is Mdm2’s partner and negatively regulates Mdm2 activity. By binding to Mdm2, p14^ARF^ increases the degradation of Mdm2, resulting in stabilization and reactivation of p53 protein. p14^ARF^-dependent stabilization of p53 is an important aspect of the function of p14^ARF^ as a tumor suppressor protein, particularly in terms of cell cycle arrest [47]. Besides promoting Mdm2 proteasomal degradation, p14^ARF^ increases nuclear localization of Mdm2, suggesting stress signals use multidirectional pathways to regulate p53 function [48]. Both in vitro and in vivo experiments have shown that the upregulation of p14^ARF^ is important for the p53-mediated stress response to activation of oncogenes [48,49,50]. While upregulation of p14^ARF^ and its binding to Mdm2 in response to oncogenic stresses suggest an important role for p14^ARF^, anticancer contributions of p14^ARF^ in human cancer are not significant based on current evidence [51]. Certainly more studies are required to understand the tumor suppressor functions of p14^ARF^ in human tumors.

While ARF’s function reverses the tumorigenic effects of Mdm2, another E3 ubiquitin ligase, Siva1, binds and ubiquitinates p14^ARF^ protein for proteasomal degradation. In fact, Siva1 is capable of promoting cell cycle progression and cell proliferation in a p14^ARF^/p53-dependent manner [52]. Recently, Park et al. reported that the Casitas B-cell lymphoma-b (Cbl-b) E3 ubiquitin ligase can target and inactivate Siva1, resulting in reactivation of p53 tumor suppressor functions in an ARF-dependent manner [53]. This finding was observed in acute myeloid leukemia. Thus, modulation of the E3 ligase activity of Cbl-b could be an effective strategy for reactivation of p53 proteins in hematologic cancers. It remains to be determined whether Cbl-b can elicit positive regulation of p53 by inhibiting Siva1 in solid tumors.

TRIM28 (Tripartite motif-containing 28), also known as KAP-1 (KRAB-associated protein 1), has several domains, including a conserved domain for RING finger B box and a leucine zipper α helical coiled-coil region. TRIM28 binds to Mdm2 through the coiled-coil domain of TRIM28 and the central acidic domain of Mdm2. By binding to Mdm2, TRIM28 promotes ubiquitination and proteasomal degradation of p53. Silencing of TRM28 promotes p53 transcriptional activity and its downstream activities, including DNA damage response and apoptosis. The above results indicate TRM28 is Mdm2’s binding partner and it negatively regulates p53 tumor suppressor functions. Interestingly, ARF proteins compete with TRIM28 in Mdm2 binding, resulting in reduction of Mdm2–TRIM28 interaction [54]. Another member of the TRIM family is TRIM13, also known as ret finger protein 2 (RFP2), which forms a complex with Mdm2 protein. By binding to Mdm2, TRIM13 increases ubiquitination and proteasomal degradation of Mdm2 in vitro and in vivo. In fact, upregulation of TRIM13 following irradiation leads to the elevation of ionizing radiation-induced apoptosis partly mediated by TRIM13-dependent stabilization of p53 [55]. TRIM19, also known as promyelocytic leukemia (PML), is a tumor suppressor protein that potentiates p53 activities. TRIM19 binds and sequesters Mdm2 protein, resulting in stabilization of p53 in the nucleus. In fact, the TRIM19/Mdm2 complex accumulates in the nucleolus in an ARF-independent manner following DNA damage. Interestingly, the presence of another protein called nucleolar protein L11 is necessary for nuclear localization of TRIM19. In the absence of nucleolar protein L11, TRIM19 is unable to localize to nucleoli and binds to Mdm2 following a DNA damage signal [56]. The cellular senescence-inhibited gene (CSIG), also known as ribosomal L1 domain-containing 1 (RSL1D1), is another nucleolar protein that binds to the Mdm2 RING finger domain and suppresses Mdm2-dependent ubiquitination and proteasomal degradation of p53 in cellular response to nucleolar stress [57]. While TRIM19 functions as a negative regulator of Mdm2 protein, a mammalian MAP (mitogen-activated protein) kinase, BMK1 (big MAP kinase 1), interferes with the interaction between PML and Mdm2, resulting in suppression of p53 activities [58].

In addition to several cytoplasmic and nuclear regulatory proteins, the p53–Mdm2 circuitry is subject to complex regulation by microRNAs (miRNAs). A good example is human hepatocellular carcinoma (HCC) where miR-122*, the complementary strand of liver-specific microRNA-122, targets and inhibits Mdm2 followed by elevated p53 protein levels and the inhibition of tumor growth in both ex vivo and animal models. Levels of miR-122* are reduced in HCC, suggesting miR-122* dominantly contributes to the tumor suppression activity mediated by p53 [59]. Pichiorri et al. completed a comprehensive study related to the role of microRNAs 192, 194, and 215 on the p53/Mdm2 autoregulatory loop. Their results indicate that p53-induced microRNAs 192, 194, and 215 lead to the degradation of Mdm2 mRNA and, consequently, significant reduction of Mdm2 protein levels. More interestingly, they showed a significant inverse correlation between miR-192 expression and Mdm2 mRNA in multiple myeloma samples. Furthermore, increased expression of microRNAs 192, 194, and 215 in multiple myeloma cells either from multiple myeloma patients or from cell lines increase the therapeutic action of MI-219 [60] or Nutlin-3a [61], two non-genotoxic activators of p53 in both in vitro and in vivo models [62].

Another inhibitor of Mdm2 is snoRNA (small nucleolar RNAs)-derived miRNA50, miR-605. The miR-605 suppresses Mdm2 through post-transcriptional repression following stress. By offsetting the Mdm2 negative-feedback loop, miR-605 allows stabilization of p53 and facilitates p53’s function in response to stress [63]. The microRNA-18b (miR-18b) is another non-coding RNA of ~22 nucleotides that functions in melanoma where miR-18b, acting as a tumor suppressor, interferes with the Mdm2–p53 pathway and reactivates p53 protein [64]. It is noteworthy that miR-18b expression induces the opposite phenotype in breast cancer cells by promoting cell proliferation, migration, and invasion [51].

In addition to Mdm2, Mdm4 is a target for miRNAs. One example is miR-191, which downregulates Mdm4 expression, resulting in p53 activation and a significant delay in ovarian carcinoma progression and tumor-related death [65]. In acute myeloid leukemia (AML) with NPM1 mutations, miR-10a positively interferes with the p53 machinery through inhibition of Mdm4 [66]. The miR-661 targets and downregulates both Mdm2 and Mdm4, resulting in elevation of p53 activity and inhibition of cell cycle progression. Depending on the status of p53 (wild-type versus mutant forms of p53), low or high levels of miR-661 expression could have opposite outcomes in different tumors [67]. The presence of miR-34a in liver cancer [68], miR-887 in prostate cancer [69], and let-7 in brain cancer [70] are other examples where miRNA can negatively regulate Mdm4. A comprehensive list of microRNAs that can regulate Mdm2 and Mdm4 was reviewed by Vijayakumaran et al. and Hoffman et al. [71,72].

Another negative regulator of Mdm2 is 14-3-3σ, which is downregulated in transformed mammary carcinoma cells. Induced by p53 following DNA damage, 14-3-3σ is involved in cell cycle checkpoint control and increases stabilization of p53 and antagonizes the tumorigenic functions of Mdm2 by blocking Mdm2-mediated p53 ubiquitination and nuclear export [73]. USP2a (ubiquitin-specific cysteine protease 2a) is a deubiquitinating enzyme that can deubiquitinate Mdm2 and positively increase Mdm2’s functions in cells. Interestingly, USP2a has no deubiquitinating activity towards p53 [74].

Similar to ARF, the ribosomal protein L11 binds to a central region in Mdm2. By binding to Mdm2, the ribosomal protein L11 (RPL11) blocks Mdm2-mediated p53 ubiquitination and degradation, resulting in activation of p53’s downstream tumor suppressor activities, including cell cycle arrest [75]. Zheng et al. showed RPL11 binds to Mdm2 and induces conformational changes in both proteins. The RPL11–Mdm2 complex blocks Mdm2-dependent inactivation of p53 in cells. By inhibiting Mdm2, RPL11 functions as a tumor suppressor protein [76]. Interestingly, PICT1 (protein interacting with carboxyl terminus 1, also known as GLTSCR2), a nucleolar protein necessary in embryogenesis events and ES cell survival, binds to RPL11. In the absence of PICT1, RPL11 is able to bind to Mdm2 and blocks Mdm2-mediated ubiquitination of p53, indicating PICT1 can be a potent regulator of the Mdm2–p53 pathway by binding and retaining RPL11 in the nucleolus. Experiments at the cellular level indicated that depletion of PICT1 in the presence of functional P53 leads to accumulation of p53 and reduction of cell growth. The tumorigenic function of PICT2 was further confirmed when Sasaki et al. found that human tumors with low expression of PICT1 present better prognoses [77].

Sui et al. showed a transcription factor Yin Yang 1 (YY1) binds directly to Mdm2 and p53. In fact, YY1 facilitates Mdm2–p53 interaction to stimulate p53 ubiquitination and degradation. Similar to other scenarios described above, the p14^ARF^ tumor suppressor protein interferes with Mdm2–YY1 interaction, reversing the negative effects of YY1 on p53 stability. As a potential cofactor for Mdm2, YY1 may have a potential role in tumorigenesis [78]. Another Mdm2 activator is AKT recruited by survival signals. It has been shown that AKT can lead to activation of Mdm2, resulting in inactivation of p53 and inhibition of p53-dependent apoptosis [79].

While Mdm2 directly targets p53, it can promote conjugation of NEDD8 (neural precursor cell expressed, developmentally down-regulated 8) to p53 resulting in NEDDylation of p53 in vitro and in vivo. NEDDylation of p53 through Mdm2–NEDD8 pathway suppresses p53’s transcriptional activity. By binding to NEDD8, Mdm2 increases its NEDDylation which can lead to Mdm2 suppression [80].

**Figure 1:**
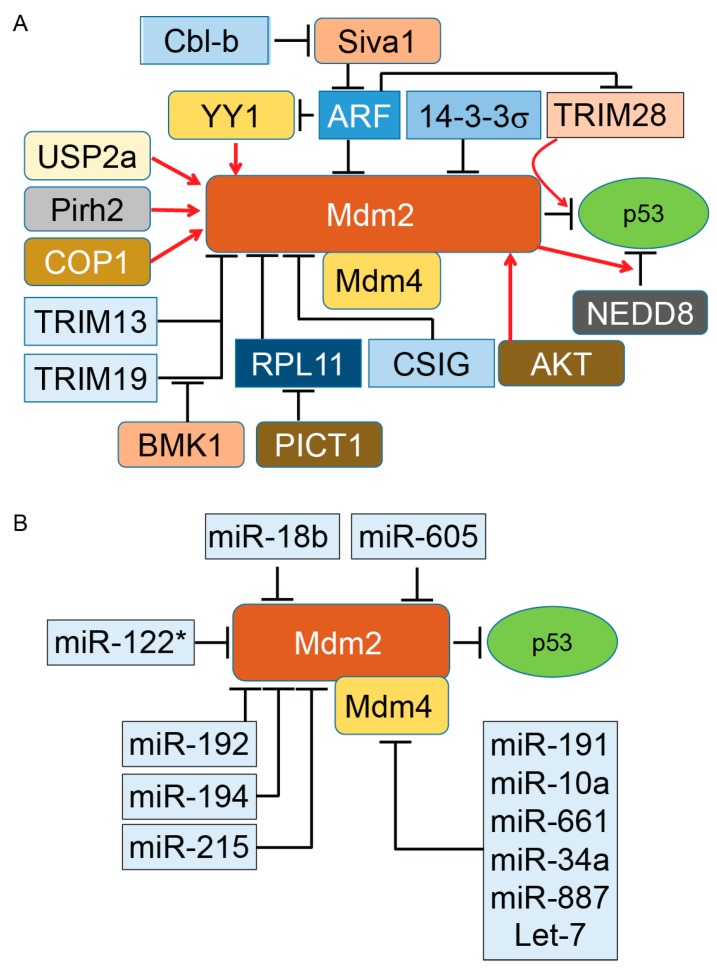
Regulation of murine double minute (Mdm)2 protein, a key E3 ubiquitin ligase of p53 tumor suppressor protein. By binding to p53, Mdm2 promotes ubiquitination of p53. The presence of Mdm4 and its binding to Mdm2 enhances ubiquitination and proteasomal degradation of p53. Basic research and clinical data indicate the important oncogenic role of both Mdm2 and Mdm4 in diverse types of tumors. However, direct inhibition of Mdm2 and Mdm4 in cancer cells has produced mixed results, particularly in animal models. The activity of either Mdm2 or Mdm4 is modulated with several regulatory proteins expressed as part of oncogenic events. Elevation or reductions of these positive and negative regulators have been reported in different cancers. Understanding the role of Mdm2’s and Mdm4’s regulators in different grades of human tumors can open a new therapeutic window for a subset of cancer patients. Panels A and B illustrate the proteins and microRNA partners of Mdm2 and Mdm4 proteins, respectively. Induction of gene expression or protein activation is shown by red arrows and inhibition of gene expression or block protein activity is shown by T-bars. Cbl-b, Casitas B-cell lymphoma-b; RPL11, ribosomal protein L11; COP1, constitutive photomorphogenesis protein 1; Pirh2, p53-induced protein with a RING-H2 domain; ARF, alternative reading frame (p14^ARF^); NEDD8, neural precursor cell expressed, developmentally down-regulated 8; USP2a, Ubiquitin-specific cysteine protease 2a; PICT1, protein interacting with carboxyl terminus 1.

Altogether, the above evidence (summarized in Figure 1A,B) indicates the complexity of Mdm2–Mdm4-dependent regulation of p53 in both physiological and pathological conditions. The above evidence indicates that multiple changes can occur in the Mdm–p53 pathway during cancer progression that positively or negatively impact patient survival. Identification of these individual factors and their alterations in different stages of tumors will certainly be beneficial for treatment strategy decisions and effective targeted therapy.

Mdm2 and its interaction with diverse protein partners (illustrated in Figure 1) indicate that the activity of Mdm2 occurs under a complex regulatory network that ultimately modulates the ubiquitination and proteasomal degradation of p53 in a cell-context-dependent manner. In addition to these partners, two other distinct regulatory mechanisms influence the Mdm2/p53 axis under normal and pathological conditions. One of these mechanisms is the protein level of Mdm2 in cells. While a low level of Mdm2 is able to monoubiquitinate p53 and induce nuclear export of p53, a high level of Mdm2 leads to p53’s polyubiquitination and nuclear degradation [20]. The second mechanism is triggered when monoubiquitinated p53 moves to the cytoplasm and recruits another set of proteins called polyubiquitin ligase (E4) enzymes [81]. The presence of E4 enzymes in the cytoplasmic compartment is essential for polyubiquitination of p53 for downstream proteasomal degradation. The p300 and CREB (cAMP-response element-binding protein)-binding protein (CBP) are two ubiquitination factors with both E3 and E4 activities required for endogenous p53 polyubiquitination and the proteasomal degradation of p53 in unstressed cells. Both p300 and CREB are exclusively cytoplasmic and absent in nuclear extracts [82,83]. Another E3 and E4 ubiquitin ligase is E4B (UBE4B), which binds to p53 and Mdm2 and promotes ubiquitination and proteasomal degradation of p53, resulting in the inhibition of p53-dependent transactivation and apoptosis. UBE4B particularly overexpresses in human brain tumors and leads to inactivation of p53 [84].

## 3. Constitutive Photomorphogenesis Protein 1 (COP1) and P53 Regulation

The E3 ubiquitin ligase constitutive COP1, also known as RFWD2 protein, is a RING finger protein. Besides the N-terminal RING finger, COP1 has an internal coiled-coil domain and WD40-repeat domains [85,86]. This E3 ligase expresses abundantly in different tissues, including tumor tissues, and contributes to development, cell survival, cell growth, and tumorigenesis [87,88,89,90,91]. COP1 binds to p53 and promotes p53 turnover by targeting it for proteasomal degradation in a ubiquitin-dependent fashion. Importantly, COP1-dependent degradation of p53 occurs independently of the Mdm2 or Pirh2 E3 ubiquitin ligases [92]. In addition, expression of wild-type COP1 or COP1 mutant lacking the RING domain in Saos-2 cells, a primary osteosarcoma cell line, showed COP1 and not the COP1ΔRING can reduce p53-dependent transactivation. In fact, overexpression of COP1 suppresses p53’s effects on cell cycle arrest and apoptosis medicated through p21 and BAX proteins, respectively [92]. Silencing of endogenous COP1 in human bone osteosarcoma epithelial cells (U2OS), a primary osteosarcoma cell line with wild-type p53 and H1299, a p53-null human non-small cell lung carcinoma, revealed the absence of COP1 arrest cell cycle in a p53-dependent manner [92]. The above evidence strongly suggests that COP1 functions as an oncogene protein in selective cancer cells, resulting in tumor progression. However, COP1 is also a dominant E3 ubiquitin ligase for c-Jun, a proto-oncogene in several cancers, including invasive breast cancer [93,94,95]. Therefore, depending on COP1’s target, this E3 ubiquitin ligase can serve as a tumor suppressor or an oncogene in different cancer types. Further experiments by Dornan et al. showed COP1 can regulate the steady-state level of p53 protein and alter p53 transactivation activity of the p21 in unstressed cells [92]. More importantly, silencing of COP1 leads to sensitization of U2OS cells to ionizing-radiation-induced cell death. Co-silencing of COP1 and Pirh2 or COP1 and Mdm2 by siRNA enhances p53 half-life 3.5-fold and 6-fold, respectively. These latter results indicate that COP1 and two other E3 ubiquitin ligases of p53, particularly Mdm2, could have a synergistic effect on p53 stability and function as well as its downstream pathways. The synergistic function of COP1 and Mdm2 on p53 may be related to the location of COP1 and Mdm2 in two different cellular compartments [92]. A set of experiments conducted in hepatocellular carcinoma (HCC) cells and an orthotopic mouse xenograft model of HCC showed that silencing of COP1 blocks HCC cell proliferation by targeting several common molecular pathways, including p53 [96]. While the above evidence highlight COP1’s tumorigenic roles in a p53-dependent manner, a set of studies completed by Migliorini et al. found COP1 has no significant effect on p53’s degradation [97]. The authors suggested that the COP1–p53 interaction may be important in certain cell types or in response to selected types of stresses [87]. More studies are needed to explain these opposing results related to the function of COP1 in cancer cells.

In another set of studies, Su et al. showed COP1 protein levels significantly decrease upon DNA damage in a 14-3-3σ-dependent manner. Significantly, 14-3-3σ promotes auto-ubiquitination of COP1 after DNA damage in an Mdm2-independent manner. The same group examined the effect of 14-3-3σ on COP1 tumorigenesis effect in a xenograft mouse model. In vivo experiments conducted by Su et al. showed that overexpression of COP1 increases tumor growth in HCT-116 colon cancer xenografts, indicating COP1 has a tumorigenic role in colon cancer through suppression of the p53 tumor suppressor pathway. More interestingly, co-expression of 14-3-3σ reverses COP1-promoted tumorigenicity in a xenograft mouse model of colon cancer [98].

**Figure 2:**
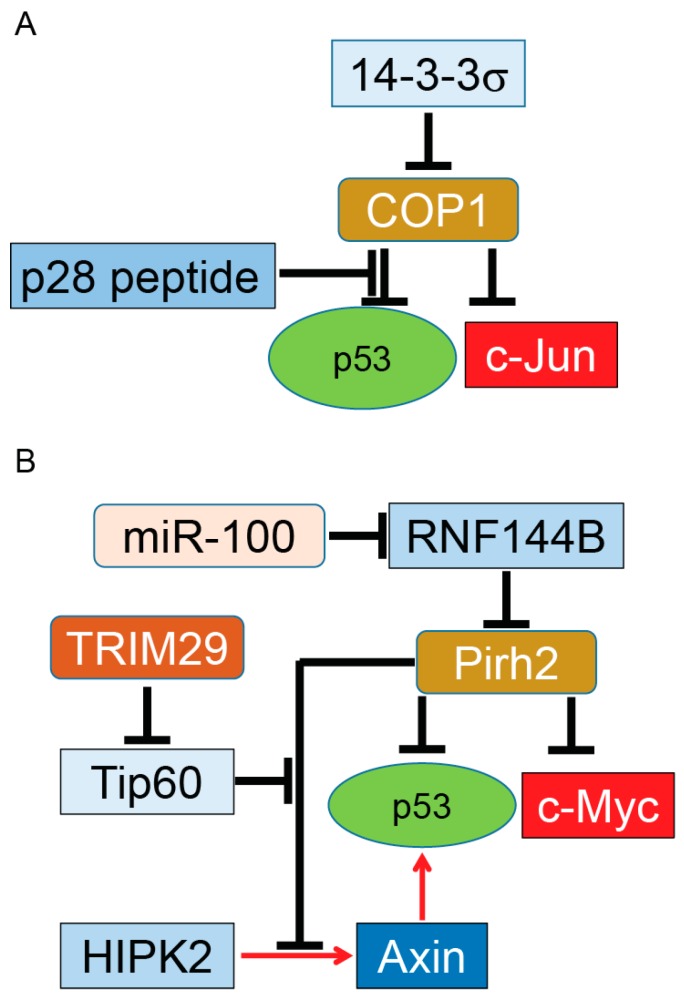
COP1 and Pirh2, tumor promoter and tumor suppressor. COP1 (**A**); and Pirh2 (**B**) serve as E3 ubiquitin ligases of p53. Overexpressed COP1 or Pirh2 can function as oncoproteins for the p53 tumor suppressor protein. On the other hand, COP1 and Pirh2 target and ubiquitinate c-Jun and c-Myc oncogenes, respectively. This dual function indicates COP1 and Pirh2 have a putative role in tumor development in tissue- and grade-dependent manners. Similar to Mdm2, COP1 and Pirh2 are regulated with their partners. It needs to be determined whether these regulators can switch the function of these two proteins from tumor suppressor to oncoprotein during tumor growth. Induction of gene expression or protein activation is shown by red arrows and inhibition of gene expression or block protein activity is shown by T-bars. Tip60, Tat-interactive protein of 60 kDa.

Overexpression of COP1 was shown in several solid tumors, including breast and ovarian cancer tissues, measured by immunohistochemistry. Overexpressed COP1 in these tumors functions as a dominant anti-p53 protein by destabilization of p53 as well as suppression of its transactivation functions. More studies are needed to determine whether targeting of COP1 or its negative regulator 14-3-3σ can have an effective therapeutic benefit in selective types of cancer (summarized in Figure 2A) [91,98]. One potential candidate is p28, a 28 amino-acid (aa) cell-penetrating peptide derived from azurin. Azurin is a redox protein secreted from the opportunistic pathogen *Pseudomonas aeruginosa* [99]. P28 binds to p53 [100] using p53’s DNA binding domain and inhibits COP1’s binding to p53, resulting in stabilization of p53 and subsequent inhibition of cancer cell growth independent of an Mdm2 pathway [101].

## 4. Pirh2 (p53-Induced Protein with a RING-H2 Domain) and p53 Regulation

Pirh2, also called ring finger and CHY (conserved cysteine and histidine involved in the binding of one zinc atom) zinc finger domain-containing 1 (Rchy1), is an E3 ubiquitin ligase that has three distinct zinc fingers: the CHY-type, the CTCHY-type (located at the C-terminus of the CHY-type), and a RING finger domain [102]. Pirh2 physically binds to p53 (residues 82–292) [102], which is distinct from Mdm2’s binding site (residues 1–51 as well as the C-terminus of p53) [103,104,105]. By binding to p53, Pirh2 promotes ubiquitination and decreases the level of p53 protein in cells. In contrast, silencing of endogenous Pirh2 expression leads to elevation of p53. The Pirh2-dependent ubiquitination and degradation of p53 suppress p53 tumor suppressor function, including transactivation and growth inhibition [102]. A set of studies using NMR spectroscopy revealed that p53 binds to both the N- and C-terminal domains of Pirh2. The C-terminal domain of Pirh2 binds to the tetramerization domain (TET) of p53, which can be enhanced by a weak interaction between the N-terminus domain of Pirh2 and the p53 DNA binding domain. By binding to the TET domain, Pirh2 preferably ubiquitinates the tetrameric form of p53 in vitro and in vivo, suggesting that Pirh2 can effectively downregulate the transcriptional active form of p53 in the cell [106].

While Pirh2 targets and ubiquitinates p53 independently of Mdm2, current evidence indicates that Pirh2 and Mdm2 could simultaneously bind to a single p53 protein and efficiently enhance its ubiquitination [46,107]. Like the Mdm2–p53 feedback mechanism, Pirh2 gene expression is regulated by p53, indicating the presence of a feedback mechanism that regulates p53 protein levels and functions [102]. It has been shown that Tip60 (Tat-interactive protein of 60 kDa) binds to Pirh2 and increases the half-life of Pirh2 protein in a COS-7 (CV-1 in Origin with SV40 genes) fibroblast-like cell line. Further studies will determine whether Tip60 can stabilize Pirh2 in cancer cells and if Tip60 alteration can change the development of tumors both in vitro and in vivo [108].

A set of in vivo experiments showed the level of p53 proteins is mildly affected in Pirh2-deficient mice. However, Pirh2 deficiency leads to higher p53 levels in response to DNA damage in several tissues. Whole-body irradiation of Pirh2^−/−^ mice or irradiation of Pirh2 knockout cells leads to elevation of p53, p53’s downstream target proteins, and apoptosis, in comparison to WT mice [109]. In addition to p53, Hakem et al. showed Pirh2 binds and mediates the ubiquitination and proteasome degradation of c-Myc, an oncoprotein frequently overexpressed in various human cancers, including lung, breast, and ovarian cancer [110]. Development of solid tumors such as sarcoma in Pirh2^+/−^ and Pirh2^−/−^ mice as well as double knockout p53^−/−^ and Pirh2^−/−^ mice indicates that Pirh2 can function as a tumor suppressor protein (summarized in Figure 2B).

While Pirh2 directly targets and inhibits p53, it also binds to the Axin–HIPK2 complex, which is involved in p53 activation through phosphorylation of p53 at Ser 46. The inhibitory effect of Pirh2 on the Axin–HIPK2 complex depends on the level of DNA damage. In cells treated with sublethal doses of doxorubicin or ultra-violet (UV) radiation, Pirh2 blocks Axin-induced p53 activation by competing with HIPK2 for binding to Axin. With a lethal dose of UV or doxorubicin, cells overexpress another regulatory protein called Tip60. Tip60 binds to Axin and suppresses formation of the Pirh2–Axin complex. A generated Axin–Tip60–HIPK2–p53 complex in the presence of a lethal dose of genotoxic factors allows full activation of p53, resulting in p53-dependent apoptosis [111]. Interestingly, Sho et al. reported that overexpression of TRIM29 enhances degradation and alters subcellular localization of Tip60, resulting in the abrogation of p53 acetylation mediated by Tip60. By suppressing p53, TRIM29 promotes cell growth, suppresses apoptosis, and triggers transforming activity. Upregulation of TRIM29 by UV irradiation suggests that TRIM29 can function as an oncogene by reversing Tip60-dependent activation of p53 [112].

A recent study completed by Yang et al. reported that a microRNA named miR-100 can enhance ubiquitination and proteasomal degradation of p53 protein in poorly differentiated gastric cancer cells while non-cancerous gastric cells remain intact. Their results indicate that knocking down miR-100 reduces the expression of Pirh2, a key E3 ubiquitin ligase for p53 ubiquitination in gastric cancer cells, resulting in stabilization and upregulation of p53 in gastric cancer cells and activation of p53’s downstream apoptosis pathway. Further studies by Yang et al showed Pirh2 is not a direct target for miR-100. In fact, miR-100 suppresses expression of RNF114B, which functions as an E3 ubiquitin ligase for Pirh2. RNF144B binds and ubiquitinates Pirh2 for proteasomal degradation. Taken together, the above studies indicate that miR-100 can indirectly trigger ubiquitination and proteasomal degradation of the p53 tumor suppressor protein in poorly differentiated gastric cancer via the miR-100–RNF144B–Pirh2–p53 pathway in both in vitro and in vivo models [113].

The ability of Pirh2 to negatively regulate the protein levels of p53 and c-Myc plus upregulation [114,115] and downregulation [109] of Pirh2 and its regulators in different types of solid tumors (summarized in Figure 2B) suggests Pirh2 may have a dual function during tumorigenesis as an oncoprotein and tumor suppressor protein in tissue-, stress-, and grade-dependent manners. The subcellular localization of Pirh2 could be another factor for its dual function, as reported for other proteins such as p27^Kip1^ [116].

## 5. Co-Chaperone Carboxyl Terminus Hsp70/90 Interacting Protein (CHIP) E3 Ubiquitin Ligase

**Figure 3:**
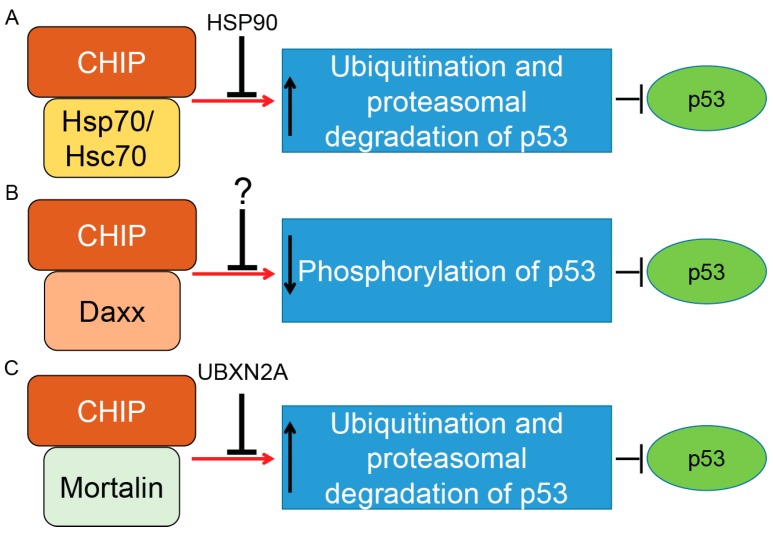
Control of p53 tumor suppressor protein by co-chaperone carboxyl terminus Hsp70/90 interacting protein (CHIP) E3 ubiquitin ligase. CHIP is a U-box-dependent E3 ubiquitin ligase. Association with chaperone proteins such as Hsp70/Hsc70 (**A**) or mitochondrial heat shock protein Mortalin-2 (**C**) allows CHIP to efficiently ubiquitinate p53 for proteasomal degradation. The present data indicate that Hsp90’s chaperone activity can inhibit CHIP/Hsp70–Hsc70–dependent degradation of p53 (wild-type and mutant forms). Thus, there is precise balance between the p53 degradation and the p53 stabilization maintained by CHIP–Hsp70–Hsc70 and Hsp90 under physiological and stress conditions. A similar inhibitory function was reported for UBXN2A protein, a ubiquitin-like (UBX) domain-containing protein, which binds and inhibits CHIP–mortalin degradation of p53 in cancer cells. CHIP also binds to the death-associated protein Daxx in a stress-dependent manner. The CHIP–Daxx interaction blocks phosphorylation of serine 46 in p53, which is necessary for activation of p53’s downstream apoptosis pathways (**B**). Blocking phosphorylation of p53 allows the CHIP–Daxx complex to interfere with the proteotoxic stress response of cells and maintain cell survival. Induction and inhibition of post-translational modification is shown by red arrows and black T-bars, respectively. Question mark indicates further research will be necessary to clarify the interactive and inhibitory effects of CHIP–Daxx complex.

CHIP (Co-Chaperone Carboxyl Terminus Hsp70/90 Interacting Protein) is a 35 kDa E3 ubiquitin ligase that has three important domains: (1) a tetratricopeptide repeat (TPR) located in the N-terminus; (2) a U-box domain located in the C-terminus; and (3) a charged coiled-coil domain located in the central of protein [117]. CHIP uses the TPR domain to interact with Hsc70–Hsp70 and Hsp90 while its C-terminal U-box domain provides E3 ubiquitin ligase activity. Through interaction with E2 enzymes of the Ubc4/5 family, CHIP induces ubiquitination and proteasomal degradation of p53 protein [118]. While overexpression of CHIP leads to proteasomal degradation of p53 and attenuation of p53-transcription activities, silencing of CHIP protein stabilizes p53 in the U2OS osteosarcoma cancer cell line [118]. CHIP induces degradation of WT-p53 and a mutant form of p53 (R175H). Cooperation of CHIP and Hsc70 is necessary for p53 degradation [118]. A molecular docking and dynamics (MD) simulation suggested that p53’s DNA binding domain is the interaction site for Hsp70 interaction [119], and mutation in this domain increases the affinity of p53 to Hsp70 in comparison to the DNA binding domain of the wild-type molecule [120]. Stankiewicz et al. confirmed that CHIP preferentially ubiquitinates Hsp70-bound p53 as one of its common substrates [121]. In fact, treatment of cells with geldanamycin, a specific inhibitor of Hsp90, enhances the degradation of WT and mutant forms of p53 [118], suggesting that association of CHIP with Hsp70 and Hsp90 has two different outcomes for p53 stability [122]. In another study, Muller et al. showed that mutant p53 proteins are preferentially associated with Hsp70 and CHIP to be ubiquitinated for proteasomal degradation in the absence of Hsp90. This study revealed a complex coordination between Hsp70, Hsp90, and CHIP when they regulate the stability of different p53 mutant proteins [122]. Positive regulation of mutant forms of p53 by Hsp90 was further confirmed when Li et al. showed that inactivation of endogenous Mdm2 and CHIP by Hsp90 leads to stabilization of mutant p53 proteins in cancer cells [123]. The relationship between Hsp90, mutant forms of p53, and CHIP became clearer when Wang et al. showed mutant p53 proteins are selectively downregulated by CHIP in cancer cells treated with an Hsp90 inhibitor, gambogic acid [124], in a ubiquitin–proteasome-dependent manner [125] (summarized in Figure 3A).

In response to stress, CHIP binds to the Daxx (death domain-associated) protein. CHIP–Daxx interaction blocks phosphorylation of serine 46 in p53, resulting in inhibition of the p53-dependent apoptotic cascade (summarized in Figure 3B) [126]. On the other hand, CHIP offers different functions in senescent cells. According to Sisoula et al., CHIP moves to the nucleus in senescent cells and leads to a significant downregulation of p53. The CHIP-dependent degradation of p53 in senescent cells is enhanced in the presence of a Hsp90 inhibitor. The authors concluded that CHIP may play a dominant role in p53 regulation during senescence [127]. Finally, it has been shown that negative regulation of p53 protein by CHIP is not specific to cancer cells. It has been reported that hypoxia-dependent downregulation of CHIP proteins leads to the accumulation of p53 in heart tissue after myocardial infarction. Targeting the p53 level and its downstream apoptotic effects through the CHIP E3 ubiquitin ligase could therefore have a therapeutic advantage for the treatment of myocardial infarction [128].

A recent finding reported by Parrales et al. shows blocking of mevalonate-5-phosphate by statins or mevalonate kinase knockdown allows CHIP to mediate nuclear export, ubiquitination, and proteasomal degradation of mutant p53. Statin or mevalonate kinase knockdown interferes with mutant p53’s binding to the Hsp40 protein DNAJA1, resulting in destabilization of mutant p53 by CHIP [129]. This study further highlights the therapeutic potential of CHIP in tumors with mutant p53 status.

Evidence in the literature indicates CHIP can function independently of Hsp90 and Hsp70 proteins. Rosser et al. showed the presence of an intrinsic chaperone activity in CHIP protein allows CHIP proteins to selectively target nonnative proteins following heat stress [130]. Tripathis et al. showed CHIP is able to restore the WT form and reduce the mutant form of p53 in cells under stress. Their results suggest that CHIP can function as a chaperone protein for WT p53 under physiological conditions as well as the resurrection of p53 mutant forms into a folded native state following cell stresses [131].

In addition to Hsp70/Hsc70 proteins, CHIP binds to another member of the Hsp70 protein family called mortalin-2, a mitochondrial Hsp70 protein and a dominant oncoprotein in several solid tumors [132]. It has been suggested that the mortalin–CHIP complex could mediate proteasomal degradation of selective substrates, including p53 [133,134]. We were able to show that UBXN2A, a ubiquitin-like ubiquitin-regulatory X (UBX) domain-containing protein, binds to mortalin-2 and releases p53 [135]. Overexpression of UBXN2A rescues downregulation of p53 in HEK293 cells overexpressing CHIP [136], indicating UBXN2A can negatively affect mortalin-2/CHIP-dependent degradation of p53 (summarized in Figure 3C).

## 6. Discussion

It has been well accepted that p53 plays an essential role in human tumorigenesis [6,137]. Current studies indicate that p53 and its network allow p53 to function beyond its primary tumor suppressor function, including metabolic regulation [138]. Dysregulation of p53 affects the hallmarks of cancer, including resisting apoptosis and sustaining cell proliferation as well as activating migration and invasion [139]. Commonly, the normal function of p53 is compromised in tumor cells as a result of somatic mutations or protein inactivation [140]. The p53 mutations are dependent on cancer type and range from 10% in hematologic cancers [141] to 50%–70% in solid tumors such as ovarian [142] and colorectal [143] cancers. Inactivation of p53 by post-translational mechanisms is responsible for almost 50% of tumors carrying WT-p53 protein [11].

**Figure 4:**
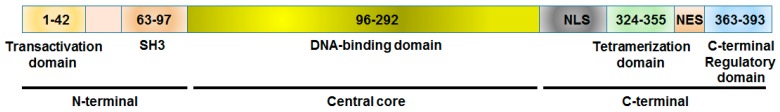
Schematic diagram showing the functional domains of the p53 tumor suppressor protein. Majority of P53 mutations found in human cancers are present in the DNA-binding domain. In addition some mutations are located in the tetramerization domain. SH3, Src homology 3 (SH3) domains; NLS, nuclear localization signal; NES, nuclear export signal.

A significant body of literature indicates that the recovery of p53’s tumor suppressor functions is a potential and effective strategy for cancer treatment [43]. The side effects generated by chemotherapeutic drugs have encouraged researchers to look for a new generation of drug that can reactivate p53 without inducing DNA damage [144]. Understanding the regulatory mechanisms involved in p53 post-translational modifications can reveal new target therapies with lower side effects and higher potency toward cancer cells. The regulation of p53 is a complex network of diverse pathways. However, it is clear that the ubiquitin–proteasome pathway is one of the main factors in p53 regulation during tumor development [145,146,147]. Further understanding the biological significance of the ubiquitin–proteasome pathway, particularly the E3 ubiquitin ligases in p53 stability and function, can lead to more accurate prognostic markers associated with the clinical course of selected tumors and the development of new drugs that can improve patients’ survival rates. In this review, we discussed four major E3 ubiquitin ligases that suppress p53 in different types of tumors (Figure 4 and Table 1).

More importantly, we highlighted the regulatory proteins that target these E3 ubiquitin ligases positively and negatively in cancer cells. Certainly, activation of p53’s E3 ubiquitin ligases and their regulatory proteins are cancer and stage dependent (Table 2). While this review provides comprehensive information about these four E3 ubiquitin ligases, further investigation is necessary to determine the level of these E3 ligases and their regulators in individual tumors in a grade-dependent manner. A complete expression profile of these E3 ligases and their regulators in patients before and after chemotherapies can open new directions for understanding the progression of tumors and the prognostic and therapeutic values of p53’s E3 ubiquitin ligases.

As direct inhibition of the proteasome complex in solid tumors failed to provide significant clinical benefit [148], a deeper understanding of the role of E3 ubiquitin ligases in the regulation of p53 will provide valuable information for the development of effective anti-cancer drugs in a specific subset of solid tumors with WT-p53.

## Figures and Tables

**Table 1:** P53’s interaction sites with the four E3 ubiquitin ligases discussed in this review. Mdm2, COP1, Pirh2 and Hsp70-CHIP distinct binding sites in the p53 protein allow these E3 ligases to often synergistically inhibit p53’s activities. * Current evidence shows that p53 uses the DNA binding domain to bind to the Hsp70–CHIP complex. The mutant form of p53 shows greater binding affinity to Hsp70. See the main text for details and related references. aa, Amino acid.

UniProt ID	Protein Symbol	Protein Names	Length (aa)	Binding Site on p53	Class of E3 Ubiquitin Ligase
Q00987	Mdm2	Mouse double minute 2 homolog (Mdm2)	491	1–51 aa and C-terminus	RING-finger containing E3 ubiqutin ligase
Q8NHY2	COP1	Constitutively photomorphogenic 1	731	Regions within the DNA-binding domain	RING-finger containing E3 ubiqutin ligase
Q96PM5	Pirh2	p53-induced RING-H2 protein	261	82–292 aa and the tetramerization domain	RING-finger containing E3 ubiqutin ligase
Q9UNE7	CHIP	C-terminus of Hsc70-interacting protein	303	DNA binding domain in p53, Hsp70 and CHIP complex *	U-box-containing E3 ubiqutin ligase

**Table 2:** This table summarizes the four major E3 ubiquitin ligases and the status of p53 (wild-type and mutant forms) discussed in this review. See the main text for details and related references. Understanding the correlations between these E3 ubiquitin ligases and the tumor suppressor functions of p53 in different types of tumors will have prognostic and therapeutic implications for a new generation of anti-cancer drugs.

E3 Ubiquitin Ligase	Human Tumor Type	P53 Status
Mmd2	Dominantly in soft tissue tumors, osteosarcomas, breast, prostate, colon cancers and esophageal carcinomas	Primarily Wild-type
Cop1	Breast adenocarcinomas, ovarian adenocarcinomas, hepatocellular carcinoma (HCC), pancreatic cancer and colorectal cancer	Primarily Wild-type
Pirh2	Hepatocellular carcinoma, clear cell renal cell carcinoma, lung neoplasms	Primarily Wild-type
CHIP	Non-small cell lung cancer and pancreatic cancer	Primarily mutant forms in tumors

## References

[1] Jenkins L.M., Durell S.R., Mazur S.J., Appella E. (2012). p53 N-terminal phosphorylation: A defining layer of complex regulation. Carcinogenesis.

[2] Weinberg R.L., Veprintsev D.B., Bycroft M., Fersht A.R. (2005). Comparative binding of p53 to its promoter and DNA recognition elements. J. Mol. Biol..

[3] Joerger A.C., Fersht A.R. (2010). The tumor suppressor p53: From structures to drug discovery. Cold Spring Harb. Perspect. Biol..

[4] Chene P. (2001). The role of tetramerization in p53 function. Oncogene.

[5] Bakalkin G., Selivanova G., Yakovleva T., Kiseleva E., Kashuba E., Magnusson K.P., Szekely L., Klein G., Terenius L., Wiman K.G. (1995). p53 binds single-stranded DNA ends through the C-terminal domain and internal DNA segments via the middle domain. Nucleic Acids Res..

[6] Bieging K.T., Mello S.S., Attardi L.D. (2014). Unravelling mechanisms of p53-mediated tumour suppression. Nat. Rev. Cancer.

[7] Vousden K.H., Prives C. (2009). Blinded by the light: The growing complexity of p53. Cell.

[8] O’Brate A., Giannakakou P. (2003). The importance of p53 location: Nuclear or cytoplasmic zip code?. Drug Resist. Updates.

[9] Ashcroft M., Taya Y., Vousden K.H. (2000). Stress signals utilize multiple pathways to stabilize p53. Mol. Cell. Biol..

[10] Inoue T., Wu L., Stuart J., Maki C.G. (2005). Control of p53 nuclear accumulation in stressed cells. FEBS Lett..

[11] Bode A.M., Dong Z. (2004). Post-translational modification of p53 in tumorigenesis. Nat. Rev. Cancer.

[12] Maki C.G. (2010). p53 Localization. p53.

[13] Lane D.P., Cheok C.F., Lain S. (2010). p53-based cancer therapy. Cold Spring Harb. Perspect. Biol..

[14] Bossi G., Sacchi A. (2007). Restoration of wild-type p53 function in human cancer: Relevance for tumor therapy. Head Neck.

[15] Zhang Q., Zeng S.X., Lu H. (2014). Targeting p53–Mdm2–Mdmx loop for cancer therapy. Subcell. Biochem..

[16] Freedman D.A., Wu L., Levine A.J. (1999). Functions of the Mdm2 oncoprotein. Cell. Mol. Life Sci..

[17] Momand J., Wu H.H., Dasgupta G. (2000). Mdm2—Master regulator of the p53 tumor suppressor protein. Gene.

[18] Wu X., Bayle J.H., Olson D., Levine A.J. (1993). The p53–Mdm2 autoregulatory feedback loop. Genes Dev..

[19] Kubbutat M.H., Jones S.N., Vousden K.H. (1997). Regulation of p53 stability by Mdm2. Nature.

[20] Li M., Brooks C.L., Wu-Baer F., Chen D., Baer R., Gu W. (2003). Mono-versus polyubiquitination: Differential control of p53 fate by Mdm2. Science.

[21] Wade M., Li Y.C., Matani A.S., Braun S.M., Milanesi F., Rodewald L.W., Wahl G.M. (2012). Functional analysis and consequences of Mdm2 E3 ligase inhibition in human tumor cells. Oncogene.

[22] Nie L., Sasaki M., Maki C.G. (2007). Regulation of p53 nuclear export through sequential changes in conformation and ubiquitination. J. Biol. Chem..

[23] Geyer R.K., Yu Z.K., Maki C.G. (2000). The Mdm2 RING-finger domain is required to promote p53 nuclear export. Nat. Cell Biol..

[24] Jones S.N., Hancock A.R., Vogel H., Donehower L.A., Bradley A. (1998). Overexpression of Mdm2 in mice reveals a p53-independent role for Mdm2 in tumorigenesis. Proc. Natl. Acad. Sci. USA.

[25] Bouska A., Eischen C.M. (2009). Murine double minute 2: p53-independent roads lead to genome instability or death. Trends Biochem. Sci..

[26] Nakayama T., Toguchida J., Wadayama B., Kanoe H., Kotoura Y., Sasaki M.S. (1995). *Mdm2* gene amplification in bone and soft-tissue tumors: Association with tumor progression in differentiated adipose-tissue tumors. Int. J. Cancer.

[27] Cordon-Cardo C., Latres E., Drobnjak M., Oliva M.R., Pollack D., Woodruff J.M., Marechal V., Chen J., Brennan M.F., Levine A.J. (1994). Molecular abnormalities of *Mdm2* and *p53* genes in adult soft tissue sarcomas. Cancer Res..

[28] Vassilev L.T. (2007). Mdm2 inhibitors for cancer therapy. Trends Mol. Med..

[29] Miller C.W., Aslo A., Won A., Tan M., Lampkin B., Koeffler H.P. (1996). Alterations of the p53, *Rb* and *Mdm2* genes in osteosarcoma. J. Cancer Res. Clin. Oncol..

[30] Reifenberger G., Liu L., Ichimura K., Schmidt E.E., Collins V.P. (1993). Amplification and overexpression of the *Mdm2* gene in a subset of human malignant gliomas without p53 mutations. Cancer Res..

[31] Watanabe T., Hotta T., Ichikawa A., Kinoshita T., Nagai H., Uchida T., Murate T., Saito H. (1994). The Mdm2 oncogene overexpression in chronic lymphocytic leukemia and low-grade lymphoma of B-cell origin. Blood.

[32] Rayburn E., Zhang R., He J., Wang H. (2005). Mdm2 and human malignancies: Expression, clinical pathology, prognostic markers, and implications for chemotherapy. Curr. Cancer Drug Targets.

[33] Burgess A., Chia K.M., Haupt S., Thomas D., Haupt Y., Lim E. (2016). Clinical overview of Mdm2/x-targeted therapies. Front. Oncol..

[34] Kojima K., Konopleva M., Samudio I.J., Shikami M., Cabreira-Hansen M., McQueen T., Ruvolo V., Tsao T., Zeng Z., Vassilev L.T. (2005). Mdm2 antagonists induce p53-dependent apoptosis in AML: Implications for leukemia therapy. Blood.

[35] Secchiero P., di Iasio M.G., Gonelli A., Zauli G. (2008). The Mdm2 inhibitor Nutlins as an innovative therapeutic tool for the treatment of haematological malignancies. Curr. Pharm. Des..

[36] Van Maerken T., Ferdinande L., Taildeman J., Lambertz I., Yigit N., Vercruysse L., Rihani A., Michaelis M., Cinatl J., Cuvelier C.A. (2009). Antitumor activity of the selective Mdm2 antagonist Nutlin-3 against chemoresistant neuroblastoma with wild-type p53. J. Natl. Cancer Inst..

[37] Secchiero P., Bosco R., Celeghini C., Zauli G. (2011). Recent advances in the therapeutic perspectives of Nutlin-3. Curr. Pharm. Des..

[38] Zhao Y., Yu H., Hu W. (2014). The regulation of Mdm2 oncogene and its impact on human cancers. Acta Biochim. Biophys. Sin..

[39] Cheok C.F., Verma C.S., Baselga J., Lane D.P. (2011). Translating p53 into the clinic. Nat. Rev. Clin. Oncol..

[40] Jones S.N., Roe A.E., Donehower L.A., Bradley A. (1995). Rescue of embryonic lethality in Mdm2-deficient mice by absence of p53. Nature.

[41] Bunderson-Schelvan M., Erbe A.K., Schwanke C., Pershouse M.A. (2009). Suppression of the mouse double minute 4 gene causes changes in cell cycle control in a human mesothelial cell line responsive to ultraviolet radiation exposure. Environ. Mol. Mutagen..

[42] Wade M., Wang Y.V., Wahl G.M. (2010). The p53 orchestra: Mdm2 and Mdmx set the tone. Trends Cell Biol..

[43] Brown C.J., Lain S., Verma C.S., Fersht A.R., Lane D.P. (2009). Awakening guardian angels: Drugging the p53 pathway. Nat. Rev. Cancer.

[44] Wang X., Wang J., Jiang X. (2011). MdmX protein is essential for Mdm2 protein-mediated p53 polyubiquitination. J. Biol. Chem..

[45] Li Q., Lozano G. (2013). Molecular pathways: Targeting Mdm2 and Mdm4 in cancer therapy. Clin. Cancer Res. Off. J. Am. Assoc. Cancer Res..

[46] Wang L., He G., Zhang P., Wang X., Jiang M., Yu L. (2011). Interplay between Mdm2, Mdmx, Pirh2 and COP1: The negative regulators of p53. Mol. Biol. Rep..

[47] Zhang Y., Xiong Y., Yarbrough W.G. (1998). ARF promotes Mdm2 degradation and stabilizes p53: ARF-INK4a locus deletion impairs both the Rb and p53 tumor suppression pathways. Cell.

[48] Sherr C.J., Weber J.D. (2000). The ARF/p53 pathway. Curr. Opin. Genet. Dev..

[49] Christophorou M.A., Ringshausen I., Finch A.J., Swigart L.B., Evan G.I. (2006). The pathological response to DNA damage does not contribute to p53-mediated tumour suppression. Nature.

[50] Efeyan A., Murga M., Martinez-Pastor B., Ortega-Molina A., Soria R., Collado M., Fernandez-Capetillo O., Serrano M. (2009). Limited role of murine ATM in oncogene-induced senescence and p53-dependent tumor suppression. PLoS ONE.

[51] Halazonetis T.D., Gorgoulis V.G., Bartek J. (2008). An oncogene-induced DNA damage model for cancer development. Science.

[52] Wang X., Zha M., Zhao X., Jiang P., Du W., Tam A.Y., Mei Y., Wu M. (2013). Siva1 inhibits p53 function by acting as an ARF E3 ubiquitin ligase. Nat. Commun..

[53] Park I.K., Blum W., Baker S.D., Caligiuri M.A. (2016). E3 ubiquitin ligase Cbl-b activates the p53 pathway by targeting Siva1, a negative regulator of ARF, in FLT3 inhibitor-resistant acute myeloid leukemia. Leukemia.

[54] Wang C., Ivanov A., Chen L., Fredericks W.J., Seto E., Rauscher F.J., Chen J. (2005). Mdm2 interaction with nuclear corepressor KAP1 contributes to p53 inactivation. EMBO J..

[55] Joo H.M., Kim J.Y., Jeong J.B., Seong K.M., Nam S.Y., Yang K.H., Kim C.S., Kim H.S., Jeong M., An S. (2011). Ret finger protein 2 enhances ionizing radiation-induced apoptosis via degradation of AKT and Mdm2. Eur. J. Cell Biol..

[56] Bernardi R., Scaglioni P.P., Bergmann S., Horn H.F., Vousden K.H., Pandolfi P.P. (2004). PML regulates p53 stability by sequestering Mdm2 to the nucleolus. Nat. Cell Biol..

[57] Xie N., Ma L., Zhu F., Zhao W., Tian F., Yuan F., Fu J., Huang D., Lv C., Tong T. (2016). Regulation of the Mdm2-p53 pathway by the nucleolar protein CSIG in response to nucleolar stress. Sci. Rep..

[58] Yang Q., Liao L., Deng X., Chen R., Gray N.S., Yates J.R., Lee J.D. (2013). BMK1 is involved in the regulation of p53 through disrupting the PML-Mdm2 interaction. Oncogene.

[59] Simerzin A., Zorde-Khvalevsky E., Rivkin M., Adar R., Zucman-Rossi J., Couchy G., Roskams T., Govaere O., Oren M., Giladi H. (2016). The liver-specific microRNA-122*, the complementary strand of microRNA-122, acts as a tumor suppressor by modulating the p53/mouse double minute 2 homolog circuitry. Hepatology.

[60] Shangary S., Qin D., McEachern D., Liu M., Miller R.S., Qiu S., Nikolovska-Coleska Z., Ding K., Wang G., Chen J. (2008). Temporal activation of p53 by a specific Mdm2 inhibitor is selectively toxic to tumors and leads to complete tumor growth inhibition. Proc. Natl. Acad. Sci. USA.

[61] Kojima K., Konopleva M., McQueen T., O’Brien S., Plunkett W., Andreeff M. (2006). Mdm2 inhibitor Nutlin-3a induces p53-mediated apoptosis by transcription-dependent and transcription-independent mechanisms and may overcome ATM-mediated resistance to fludarabine in chronic lymphocytic leukemia. Blood.

[62] Pichiorri F., Suh S.-S., Rocci A., De Luca L., Taccioli C., Santhanam R., Zhou W., Benson D.M., Hofmainster C., Alder H. (2010). Downregulation of p53-inducible microRNAs 192, 194, and 215 Impairs the p53/Mdm2 Autoregulatory Loop in Multiple Myeloma Development. Cancer Cell.

[63] Xiao J., Lin H., Luo X., Luo X., Wang Z. (2011). miR-605 joins p53 network to form a p53:miR-605:Mdm2 positive feedback loop in response to stress. EMBO J..

[64] Dar A.A., Majid S., Rittsteuer C., de Semir D., Bezrookove V., Tong S., Nosrati M., Sagebiel R., Miller J.R., Kashani-Sabet M. (2013). The role of miR-18b in Mdm2-p53 pathway signaling and melanoma progression. J. Natl. Cancer Inst..

[65] Wynendaele J., Bohnke A., Leucci E., Nielsen S.J., Lambertz I., Hammer S., Sbrzesny N., Kubitza D., Wolf A., Gradhand E. (2010). An illegitimate microRNA target site within the 3′ UTR of Mdm4 affects ovarian cancer progression and chemosensitivity. Cancer Res..

[66] Ovcharenko D., Stolzel F., Poitz D., Fierro F., Schaich M., Neubauer A., Kelnar K., Davison T., Muller-Tidow C., Thiede C. (2011). miR-10a overexpression is associated with NPM1 mutations and Mdm4 downregulation in intermediate-risk acute myeloid leukemia. Exp. Hematol..

[67] Hoffman Y., Bublik D.R., Pilpel Y., Oren M. (2014). miR-661 downregulates both Mdm2 and Mdm4 to activate p53. Cell Death Differ..

[68] Mandke P., Wyatt N., Fraser J., Bates B., Berberich S.J., Markey M.P. (2012). MicroRNA-34a modulates Mdm4 expression via a target site in the open reading frame. PLoS ONE.

[69] Stegeman S., Moya L., Selth L.A., Spurdle A.B., Clements J.A., Batra J. (2015). A genetic variant of Mdm4 influences regulation by multiple microRNAs in prostate cancer. Endocr. Relat. Cancer.

[70] Xie C., Chen W., Zhang M., Cai Q., Xu W., Li X., Jiang S. (2015). Mdm4 regulation by the let-7 miRNA family in the DNA damage response of glioma cells. FEBS Lett..

[71] Vijayakumaran R., Tan K.H., Miranda P.J., Haupt S., Haupt Y. (2015). Regulation of mutant p53 protein expression. Front. Oncol..

[72] Hoffman Y., Pilpel Y., Oren M. (2014). microRNAs and Alu elements in the p53–Mdm2–Mdm4 regulatory network. J. Mol. Cell Biol..

[73] Yang H.Y., Wen Y.Y., Chen C.H., Lozano G., Lee M.H. (2003). 14-3-3σ positively regulates p53 and suppresses tumor growth. Mol. Cell. Biol..

[74] Stevenson L.F., Sparks A., Allende-Vega N., Xirodimas D.P., Lane D.P., Saville M.K. (2007). The deubiquitinating enzyme USP2a regulates the p53 pathway by targeting Mdm2. EMBO J..

[75] Zhang Y., Wolf G.W., Bhat K., Jin A., Allio T., Burkhart W.A., Xiong Y. (2003). Ribosomal protein L11 negatively regulates oncoprotein Mdm2 and mediates a p53-dependent ribosomal-stress checkpoint pathway. Mol. Cell. Biol..

[76] Zheng J., Lang Y., Zhang Q., Cui D., Sun H., Jiang L., Chen Z., Zhang R., Gao Y., Tian W. (2015). Structure of human Mdm2 complexed with RPL11 reveals the molecular basis of p53 activation. Genes Dev..

[77] Sasaki M., Kawahara K., Nishio M., Mimori K., Kogo R., Hamada K., Itoh B., Wang J., Komatsu Y., Yang Y.R. (2011). Regulation of the Mdm2–P53 pathway and tumor growth by PICT1 via nucleolar RPL11. Nat. Med..

[78] Sui G., Affar el B., Shi Y., Brignone C., Wall N.R., Yin P., Donohoe M., Luke M.P., Calvo D., Grossman S.R. (2004). Yin Yang 1 is a negative regulator of p53. Cell.

[79] Gottlieb T.M., Leal J.F., Seger R., Taya Y., Oren M. (2002). Cross-talk between Akt, p53 and Mdm2: Possible implications for the regulation of apoptosis. Oncogene.

[80] Xirodimas D.P., Saville M.K., Bourdon J.C., Hay R.T., Lane D.P. (2004). Mdm2-mediated NEDD8 conjugation of p53 inhibits its transcriptional activity. Cell.

[81] Koegl M., Hoppe T., Schlenker S., Ulrich H.D., Mayer T.U., Jentsch S. (1999). A novel ubiquitination factor, E4, is involved in multiubiquitin chain assembly. Cell.

[82] Shi D., Pop M.S., Kulikov R., Love I.M., Kung A.L., Grossman S.R. (2009). CBP and p300 are cytoplasmic E4 polyubiquitin ligases for p53. Proc. Natl. Acad. Sci. USA.

[83] Grossman S.R., Deato M.E., Brignone C., Chan H.M., Kung A.L., Tagami H., Nakatani Y., Livingston D.M. (2003). Polyubiquitination of p53 by a ubiquitin ligase activity of p300. Science.

[84] Wu H., Pomeroy S.L., Ferreira M., Teider N., Mariani J., Nakayama K.I., Hatakeyama S., Tron V.A., Saltibus L.F., Spyracopoulos L. (2011). UBE4B promotes Hdm2-mediated degradation of the tumor suppressor p53. Nat. Med..

[85] Deng X.W., Caspar T., Quail P.H. (1991). cop1: A regulatory locus involved in light-controlled development and gene expression in Arabidopsis. Genes Dev..

[86] Bianchi E., Denti S., Catena R., Rossetti G., Polo S., Gasparian S., Putignano S., Rogge L., Pardi R. (2003). Characterization of human constitutive photomorphogenesis protein 1, a RING finger ubiquitin ligase that interacts with Jun transcription factors and modulates their transcriptional activity. J. Biol. Chem..

[87] Marine J.-C. (2012). Spotlight on the role of COP1 in tumorigenesis. Nat. Rev. Cancer.

[88] Li Y.F., Wang D.D., Zhao B.W., Wang W., Huang C.Y., Chen Y.M., Zheng Y., Keshari R.P., Xia J.C., Zhou Z.W. (2012). High level of COP1 expression is associated with poor prognosis in primary gastric cancer. Int. J. Biol. Sci..

[89] Li J., Wang L., Xiao R., Pan Q., Huang H., Kuang R. (2016). High expression of constitutive photomorphogenic 1 (COP1) is associated with poor prognosis in bladder cancer. Tumour Biol..

[90] Zou S., Zhu Y., Wang B., Qian F., Zhang X., Wang L., Fu C., Bao H., Xie M., Gao S. (2016). The ubiquitin ligase COP1 promotes glioma cell proliferation by preferentially downregulating tumor suppressor p53. Mol. Neurobiol..

[91] Dornan D., Bheddah S., Newton K., Ince W., Frantz G.D., Dowd P., Koeppen H., Dixit V.M., French D.M. (2004). COP1, the negative regulator of p53, is overexpressed in breast and ovarian adenocarcinomas. Cancer Res..

[92] Dornan D., Wertz I., Shimizu H., Arnott D., Frantz G.D., Dowd P., O’Rourke K., Koeppen H., Dixit V.M. (2004). The ubiquitin ligase COP1 is a critical negative regulator of p53. Nature.

[93] Vleugel M.M., Greijer A.E., Bos R., van der Wall E., van Diest P.J. (2006). c-Jun activation is associated with proliferation and angiogenesis in invasive breast cancer. Hum. Pathol..

[94] Wertz I.E., O’Rourke K.M., Zhang Z., Dornan D., Arnott D., Deshaies R.J., Dixit V.M. (2004). Human De-etiolated-1 regulates c-Jun by assembling a CUL4A ubiquitin ligase. Science.

[95] Shao J., Teng Y., Padia R., Hong S., Noh H., Xie X., Mumm J.S., Dong Z., Ding H.F., Cowell J. (2013). COP1 and GSK3β cooperate to promote c-Jun degradation and inhibit breast cancer cell tumorigenesis. Neoplasia.

[96] Lee Y.H., Andersen J.B., Song H.T., Judge A.D., Seo D., Ishikawa T., Marquardt J.U., Kitade M., Durkin M.E., Raggi C. (2010). Definition of ubiquitination modulator COP1 as a novel therapeutic target in human hepatocellular carcinoma. Cancer Res..

[97] Migliorini D., Bogaerts S., Defever D., Vyas R., Denecker G., Radaelli E., Zwolinska A., Depaepe V., Hochepied T., Skarnes W.C. (2011). COP1 constitutively regulates c-Jun protein stability and functions as a tumor suppressor in mice. J. Clin. Investig..

[98] Su C.H., Zhao R., Zhang F., Qu C., Chen B., Feng Y.H., Phan L., Chen J., Wang H., Wang H. (2011). 14-3-3σ exerts tumor-suppressor activity mediated by regulation of COP1 stability. Cancer Res..

[99] Jia L., Gorman G.S., Coward L.U., Noker P.E., McCormick D., Horn T.L., Harder J.B., Muzzio M., Prabhakar B., Ganesh B. (2011). Preclinical pharmacokinetics, metabolism, and toxicity of azurin-p28 (NSC745104) a peptide inhibitor of p53 ubiquitination. Cancer Chemother. Pharmacol..

[100] Apiyo D., Wittung-Stafshede P. (2005). Unique complex between bacterial azurin and tumor-suppressor protein p53. Biochem. Biophys. Res. Commun..

[101] Yamada T., Christov K., Shilkaitis A., Bratescu L., Green A., Santini S., Bizzarri A.R., Cannistraro S., Gupta T.K., Beattie C.W. (2013). p28, a first in class peptide inhibitor of cop1 binding to p53. Br. J. Cancer.

[102] Leng R.P., Lin Y., Ma W., Wu H., Lemmers B., Chung S., Parant J.M., Lozano G., Hakem R., Benchimol S. (2003). Pirh2, a p53-induced ubiquitin-protein ligase, promotes p53 degradation. Cell.

[103] Chen J., Marechal V., Levine A.J. (1993). Mapping of the p53 and Mdm-2 interaction domains. Mol. Cell. Biol..

[104] Picksley S.M., Vojtesek B., Sparks A., Lane D.P. (1994). Immunochemical analysis of the interaction of p53 with Mdm2—Fine mapping of the Mdm2 binding site on p53 using synthetic peptides. Oncogene.

[105] Poyurovsky M.V., Katz C., Laptenko O., Beckerman R., Lokshin M., Ahn J., Byeon I.J., Gabizon R., Mattia M., Zupnick A. (2010). The C terminus of p53 binds the N-terminal domain of Mdm2. Nat. Struct. Mol. Biol..

[106] Sheng Y., Laister R., Lemak A., Wu B., Tai E., Duan S., Lukin J., Sunnerhagen M., Srisailam S., Karra M. (2008). Molecular basis of Pirh2-mediated p53 ubiquitylation. Nat. Struct. Mol. Biol..

[107] Yang Y., Li C.-C.H., Weissman A.M. (2004). Regulating the p53 system through ubiquitination. Oncogene.

[108] Logan I.R., Sapountzi V., Gaughan L., Neal D.E., Robson C.N. (2004). Control of human PIRH2 protein stability: Involvement of Tip60 and the proteasome. J. Biol. Chem..

[109] Hakem A., Bohgaki M., Lemmers B., Tai E., Salmena L., Matysiak-Zablocki E., Jung Y.S., Karaskova J., Kaustov L., Duan S. (2011). Role of Pirh2 in mediating the regulation of p53 and c-Myc. PLoS Genet..

[110] Meyer N., Penn L.Z. (2008). Reflecting on 25 years with MYC. Nat. Rev. Cancer.

[111] Li Q., Lin S., Wang X., Lian G., Lu Z., Guo H., Ruan K., Wang Y., Ye Z., Han J. (2009). Axin determines cell fate by controlling the p53 activation threshold after DNA damage. Nat. Cell Biol..

[112] Sho T., Tsukiyama T., Sato T., Kondo T., Cheng J., Saku T., Asaka M., Hatakeyama S. (2011). TRIM29 negatively regulates p53 via inhibition of Tip60. Biochim. Biophys. Acta.

[113] Yang G., Gong Y., Wang Q., Wang L., Zhang X. (2016). miR-100 antagonism triggers apoptosis by inhibiting ubiquitination-mediated p53 degradation. Oncogene.

[114] Logan I.R., Gaughan L., McCracken S.R., Sapountzi V., Leung H.Y., Robson C.N. (2006). Human PIRH2 enhances androgen receptor signaling through inhibition of histone deacetylase 1 and is overexpressed in prostate cancer. Mol. Cell. Biol..

[115] Duan W., Gao L., Druhan L.J., Zhu W.G., Morrison C., Otterson G.A., Villalona-Calero M.A. (2004). Expression of Pirh2, a newly identified ubiquitin protein ligase, in lung cancer. J. Natl. Cancer Inst..

[116] Kossatz U., Malek N.P. (2007). p27: Tumor suppressor and oncogene...?. Cell Res..

[117] McDonough H., Patterson C. (2003). CHIP: A link between the chaperone and proteasome systems. Cell Stress Chaperones.

[118] Esser C., Scheffner M., Hohfeld J. (2005). The chaperone-associated ubiquitin ligase CHIP is able to target p53 for proteasomal degradation. J. Biol. Chem..

[119] Elengoe A., Naser M.A., Hamdan S. (2015). A novel protein interaction between nucleotide binding domain of Hsp70 and p53 motif. Int. J. Genom..

[120] Fourie A.M., Hupp T.R., Lane D.P., Sang B.C., Barbosa M.S., Sambrook J.F., Gething M.J.H. (1997). Hsp70 binding sites in the tumor suppressor protein p53. J. Biol. Chem..

[121] Stankiewicz M., Nikolay R., Rybin V., Mayer M.P. (2010). CHIP participates in protein triage decisions by preferentially ubiquitinating Hsp70-bound substrates. FEBS J..

[122] Muller P., Hrstka R., Coomber D., Lane D.P., Vojtesek B. (2008). Chaperone-dependent stabilization and degradation of p53 mutants. Oncogene.

[123] Li D., Marchenko N.D., Schulz R., Fischer V., Velasco-Hernandez T., Talos F., Moll U.M. (2011). Functional inactivation of endogenous Mdm2 and CHIP by HSP90 causes aberrant stabilization of mutant p53 in human cancer cells. Mol. Cancer Res..

[124] Davenport J., Manjarrez J.R., Peterson L., Krumm B., Blagg B.S., Matts R.L. (2011). Gambogic acid, a natural product inhibitor of Hsp90. J. Nat. Prod..

[125] Wang J., Zhao Q., Qi Q., Gu H.Y., Rong J.J., Mu R., Zou M.J., Tao L., You Q.D., Guo Q.L. (2011). Gambogic acid-induced degradation of mutant p53 is mediated by proteasome and related to CHIP. J. Cell. Biochem..

[126] McDonough H., Charles P.C., Hilliard E.G., Qian S.B., Min J.N., Portbury A., Cyr D.M., Patterson C. (2009). Stress-dependent Daxx-CHIP interaction suppresses the p53 apoptotic program. J. Biol. Chem..

[127] Sisoula C., Trachana V., Patterson C., Gonos E.S. (2011). CHIP-dependent p53 regulation occurs specifically during cellular senescence. Free Radic. Biol. Med..

[128] Naito A.T., Okada S., Minamino T., Iwanaga K., Liu M.L., Sumida T., Nomura S., Sahara N., Mizoroki T., Takashima A. (2010). Promotion of CHIP-mediated p53 degradation protects the heart from ischemic injury. Circ. Res..

[129] Parrales A., Ranjan A., Iyer S.V., Padhye S., Weir S.J., Roy A., Iwakuma T. (2016). DNAJA1 controls the fate of misfolded mutant p53 through the mevalonate pathway. Nat. Cell Biol..

[130] Rosser M.F., Washburn E., Muchowski P.J., Patterson C., Cyr D.M. (2007). Chaperone functions of the E3 ubiquitin ligase CHIP. J. Biol. Chem..

[131] Tripathi V., Ali A., Bhat R., Pati U. (2007). CHIP chaperones wild type p53 tumor suppressor protein. J. Biol. Chem..

[132] Black J.D., Rezvani K. (2016). Heat shock protein 70s as potential molecular targets for colon cancer therapeutics. Curr. Med. Chem..

[133] Kaul S.C., Deocaris C.C., Wadhwa R. (2007). Three faces of mortalin: A housekeeper, guardian and killer. Exp. Gerontol..

[134] Wadhwa R., Ryu J., Ahn H.M., Saxena N., Chaudhary A., Yun C.O., Kaul S.C. (2015). Functional significance of point mutations in stress chaperone mortalin and their relevance to Parkinson disease. J. Biol. Chem..

[135] Sane S., Abdullah A., Boudreau D.A., Autenried R.K., Gupta B.K., Wang X., Wang H., Schlenker E.H., Zhang D., Telleria C. (2014). Ubiquitin-like (UBX)-domain-containing protein, UBXN2A, promotes cell death by interfering with the p53-Mortalin interactions in colon cancer cells. Cell Death Dis..

[136] Sane S., Abdullah A., Nelson M.E., Wang H., Chauhan S.C., Newton S.S., Rezvani K. (2016). Structural studies of UBXN2A and mortalin interaction and the putative role of silenced UBXN2A in preventing response to chemotherapy. Cell Stress Chaperones.

[137] Levine A.J., Oren M. (2009). The first 30 years of p53: Growing ever more complex. Nat. Rev. Cancer.

[138] Vousden K.H., Ryan K.M. (2009). p53 and metabolism. Nat. Rev. Cancer.

[139] Vogelstein B., Lane D., Levine A.J. (2000). Surfing the p53 network. Nature.

[140] Hollstein M., Sidransky D., Vogelstein B., Harris C.C. (1991). p53 mutations in human cancers. Science.

[141] Peller S., Rotter V. (2003). TP53 in hematological cancer: Low incidence of mutations with significant clinical relevance. Hum. Mutat..

[142] Schuijer M., Berns E.M. (2003). TP53 and ovarian cancer. Hum. Mutat..

[143] Iacopetta B. (2003). TP53 mutation in colorectal cancer. Hum. Mutat..

[144] Lain S., Lane D. (2003). Improving cancer therapy by non-genotoxic activation of p53. Eur. J. Cancer.

[145] Jain A.K., Barton M.C. (2010). Making sense of ubiquitin ligases that regulate p53. Cancer Biol. Ther..

[146] Brooks C.L., Gu W. (2011). p53 regulation by ubiquitin. FEBS Lett..

[147] Lee J.T., Gu W. (2010). The multiple levels of regulation by p53 ubiquitination. Cell Death Differ..

[148] Mackay H., Hedley D., Major P., Townsley C., Mackenzie M., Vincent M., Degendorfer P., Tsao M.S., Nicklee T., Birle D. (2005). A phase II trial with pharmacodynamic endpoints of the proteasome inhibitor bortezomib in patients with metastatic colorectal cancer. Clin. Cancer Res..

